# The Role of Lipid Sensing Nuclear Receptors (PPARs and LXR) and Metabolic Lipases in Obesity, Diabetes and NAFLD

**DOI:** 10.3390/genes12050645

**Published:** 2021-04-26

**Authors:** Emmanuel D. Dixon, Alexander D. Nardo, Thierry Claudel, Michael Trauner

**Affiliations:** Hans Popper Laboratory of Molecular Hepatology, Department of Internal Medicine III, Division of Gastroenterology and Hepatology, Medical University of Vienna, 1090 Vienna, Austria; emmanuel.dixon@meduniwien.ac.at (E.D.D.); alexander.nardo@gmail.com (A.D.N.); thierry.claudel@meduniwien.ac.at (T.C.)

**Keywords:** nuclear receptor, NAFLD, metabolic lipase, obesity, diabetes

## Abstract

Obesity and type 2 diabetes mellitus (T2DM) are metabolic disorders characterized by metabolic inflexibility with multiple pathological organ manifestations, including non-alcoholic fatty liver disease (NAFLD). Nuclear receptors are ligand-dependent transcription factors with a multifaceted role in controlling many metabolic activities, such as regulation of genes involved in lipid and glucose metabolism and modulation of inflammatory genes. The activity of nuclear receptors is key in maintaining metabolic flexibility. Their activity depends on the availability of endogenous ligands, like fatty acids or oxysterols, and their derivatives produced by the catabolic action of metabolic lipases, most of which are under the control of nuclear receptors. For example, adipose triglyceride lipase (ATGL) is activated by peroxisome proliferator-activated receptor γ (PPARγ) and conversely releases fatty acids as ligands for PPARα, therefore, demonstrating the interdependency of nuclear receptors and lipases. The diverse biological functions and importance of nuclear receptors in metabolic syndrome and NAFLD has led to substantial effort to target them therapeutically. This review summarizes recent findings on the roles of lipases and selected nuclear receptors, PPARs, and liver X receptor (LXR) in obesity, diabetes, and NAFLD.

## 1. Introduction

The concept of metabolic flexibility describes the ability to switch between the two predominant sources of energy, carbohydrates and lipids [1]. Postprandial elevation of blood glucose stimulates the pancreatic β cells to release insulin, which simultaneously induces glucose uptake and inhibits lipolysis in metabolic tissues. In the fasted state, however, a counter-regulatory hormonal network ensures a normal blood glucose level through hepatic glycogen catabolism and gluconeogenesis while concurrently inhibiting insulin release with activation of tissue lipolysis. The metabolic flexibility was a survival-evolutionary strategy adopted by early humans during pre-agricultural evolution (Neolithic hunters and gatherer era) when food availability was scarce [1]. The bountiful nutrients in modern times have inevitably modified many evolutionary-based behavior patterns in humans leading to the disruption of metabolic flexibility and resulting in developing metabolic syndrome. Metabolic syndrome is a sequence of interrelated metabolic disorders, including obesity, insulin resistance (IR), developing type 2 diabetes mellitus (T2DM), dyslipidemia, hypertension, and atherosclerosis. Obesity and T2DM are a continuum of metabolic disorders with divergent pathological manifestations and often lead to non-alcoholic fatty liver disease (NAFLD) [2,3].

The metabolic syndrome is attributed to an underlying impairment of glucose and lipid metabolism in adipose tissue and the liver [2,3], neither of which have evolved adequately to cope with the continuous chronic oversupply of nutrients often seen in the obese state. Accumulation of fat causes hypertrophy and hypoxia in the adipose tissue, which elicit an immune response to restore homeostasis. However, this response is maladaptive and often leads to IR and the loss of metabolic flexibility [4].

At the cellular level, metabolic flexibility is achieved through energy sensors, such as nuclear receptors, that either activate, inhibit, or trans-repress specific metabolic pathways. The activation of nuclear receptors is enabled by endogenous ligands, obtained by the catabolic action of metabolic lipases. Therefore, nuclear receptors and lipases are indispensable in commandeering metabolic signaling pathways involved in regulating energy balance, and their abnormal signaling plays a key role in developing metabolic syndrome and NAFLD (reviewed in [5]). In this review, we summarize recent findings on the roles of metabolic lipases and selected nuclear receptors, peroxisome proliferator-activated receptors (PPARs), and liver X receptor (LXR) in obesity, diabetes, and NAFLD.

## 2. The Characterization and Identification of the PPARs and LXRs Nuclear Receptors

Nuclear receptors are soluble receptors belonging to the superfamily of ligand-regulated transcription factors. Nuclear receptors localize primarily in the nucleus and can be activated by endogenous lipid-soluble ligands, namely fatty acids (FAs) and their derivatives, retinoic acids, oxysterol, thyroid hormones, bile acids, and steroid hormones, as well as synthetic or exogenous ligands (reviewed in [5]). Their activation regulates the expression of several genes involved in various biological processes, such as metabolism.

### 2.1. Peroxisome Proliferator-Activated Receptors (PPARs)

PPARs regulate various genes involved in virtually all pathways of lipid and glucose metabolism in metabolic tissues, such as the adipose tissue and the liver [6,7]. Three PPAR isotypes have been identified, namely PPARα (NR1C1), PPAR-β/δ (NR1C2), and PPARγ (NR1C3), each encoded by a unique gene and displaying isoform-specific tissue distribution pattern and function. PPARα is mainly expressed in tissues with a high rate of FA oxidation, such as the liver, heart, skeletal muscle, brown adipose tissues (BAT), kidney, and to a lesser extent, the white adipose tissues (WAT). PPAR-β/δ is expressed in the liver, skeletal muscle, macrophages, adipose tissues, lungs, brain, and skin [8]. The three PPARγ isoforms, γ1, γ2, and γ3, localize in different tissues; PPARγ1 is ubiquitously expressed, whereas PPARγ2 is mainly found in adipose tissues, and PPARγ3 is the most abundant isoform in macrophages, colon, and adipose tissues [5]. Although they share a high degree of homology, they differ in ligand specificity (reviewed in [9]).

**Figure 1:**
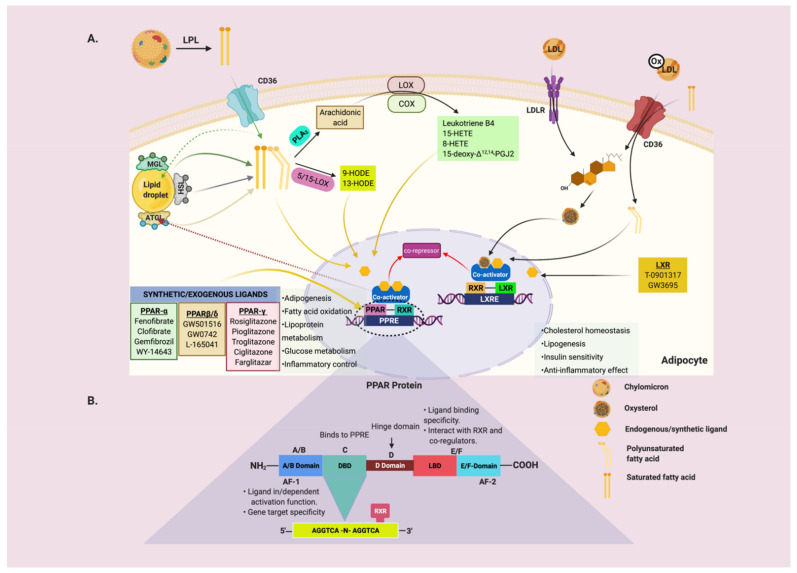
(**A**) General mechanisms of the interdependency between lipid sensing nuclear receptors and metabolic lipases. Dietary FAs are re-esterified into TAG and secreted as part of chylomicron. Plasma chylomicron undergoes rapid hydrolytic activities by LPL. The FAs produced are taken up by the adipose tissues and other underlying tissues via the FAs transporter, CD36. The assimilated FAs are either stored in lipid droplets, undergo oxidation, or as ligands for the lipid sensing nuclear receptors, such as PPARs and LXR. When required, the lipid droplets release FAs through the catabolic actions of ATGL regulated by PPARγ. The FAs and their enzymatically derived derivatives, such as 15-HETE, 8-HETE, 15d-PGJ2, 9-HODE, 13-HODE, LTB4, and exogenous ligands, are potent endogenous ligands for the PPARs. Oxysterols are ligands for LXR/RXR. The liganded PPAR/RXR heterodimer causes a conformational change that leads to the displacement of the corepressor and recruits’ coactivators to drive transcriptional activities. (**B**) Schematic representation of the PPAR: The PPAR structure comprises six domains, namely A/B, DBD, D-domain, LBD, and E/F domain. When PPAR is liganded and heterodimerizes with RXR, these domains integrate intracellular signals to direct the transcriptional activity of multiple target genes through their PPRE that harbors in their promoter regions. Abbreviations: ATGL: adipose triglyceride lipase, AF-1/2: activating factor 1, 2, COX: cyclooxygenase, DBD: DNA-binding domain, FFA: free fatty acids, HETE: hydroxyeicosatetraenoic acid, HODE: hydroxyoctadeca-9Z,11E-dienoic acids, HSL: hormone-sensitive lipase, LBD: ligand-binding domain, LOX: lipoxygenase, LPL: lipoprotein lipase, LTB4: leukotriene B4, LXR: liver X receptor, MGL: monoglyceride lipase, PPAR: peroxisome proliferator-activated receptor, PPRE: PPAR response element, 15d-PGJ2:15-deoxy-Δ12,14-prostaglandin J2, RXR: retinoid X receptor. Created with BioRender.com, accessed on 16 March 2021.

To become transcriptionally active, PPARs heterodimerizes with the retinoid X receptors (RXRs) (NR2B1-3) [10] and binds to a specific DNA sequence known as peroxisome proliferator response element (PPRE) either in the enhancer or the promoter region of the regulated genes (Figure 1B). Ablation of PPARγ and/or RXRα in mice resulted in impaired adipogenesis and lipolysis and increased lipoprotein lipase activity in skeletal muscles than wild-type (WT) mice [11]. PPARs have the classical six domains architecture, namely A/B domain, DNA-binding domain (DBD), hinge domain (D-domain), ligand-binding domain (LBD), and E/F domain (Figure 1B). These domains integrate intracellular signals to control the transcriptional activity of multiple target genes [12]. The A/B domain harbors the transcriptional activation 1 (AF-1) region that is a determinant of isoform-specific target gene activation and responsible for basal, ligand-binding independent, and dependent activity [12]. The A/B domain linked to the DBD (domain C) contains two zinc-fingers that bind the PPREs. The response element for PPAR consists of a direct repeat of six nucleotides separated by a single base pair (AGGTCA-n-AGGTCA), forming the so-called direct repeat 1 (DR1). The 5′ flanking nucleotides of the core PPRE play a crucial role in PPAR subtype specificity since PPAR interacts with the 5′ motif, while RXR binds to the downstream 3′ [13]. The hinge region is a highly flexible domain linking the DBD and the LBD. Ligand-dependent transcriptional activation of nuclear receptors largely relies on a highly conserved motif in the LBD, referred to as AF-2 [10].

Transcriptional regulation by PPAR/RXR heterodimers depends on binding a cognate PPAR or RXR ligand [10]. Indeed, ligand-activated PPAR/RXR undergoes a conformational change that catalyzes the displacement of corepressors and recruits co-factors to the promoter region of the targeted genes to initiate transcription (Figure 1A). The typical endogenous ligand for PPARs are FAs and their derivatives like the eicosanoids, prostaglandins, and leukotriene B4 (Table 1) (reviewed in ([5]). Several synthetic PPAR ligands also exist, for example; fibrates and Wy-14643 (specific PPARα activators) [14,15], GW501516 (specific PPAR-β/δ activator) [5], and thiazolidinediones (TZD) derivatives (troglitazone, pioglitazone, GW1929, and GW2090) are specific PPARγ activators (reviewed in [16,17,18,19]). The PPARγ agonists rosiglitazone and pioglitazone were shown to improve NAFLD-related features of hepatic steatosis, ballooning, inflammation, and in several studies, also stage of fibrosis in non-diabetic, prediabetic and T2DM patients with NAFLD [20,21,22,23]. Interestingly, RXR and PPARγ antagonism was reported to ameliorate high-fat diet (HFD)-induced obesity and IR through reduction of triacylglycerol (TAG) in WAT, skeletal muscle, and liver in KKAy mice (a genetic model for obesity-diabetes syndrome) in contrast with untreated mice [24]. However, RXR and PPARγ antagonism caused the reemergence of IR in heterozygous PPARγ deficient mice [24]. Another study showed that PPARγ deficiency protected mice from HFD-induced adipocyte hypertrophy, obesity, and IR [25]. In line with this study, the Pro12Ala polymorphism in human PPARγ that moderately reduces the transcriptional activity of PPARγ and confers resistance to T2DM [26].

At variance with the canonical transcriptional modulation of metabolic processes, PPAR anti-inflammatory functions are carried out through an alternative mechanism, defined as receptor-dependent trans-repression. The majority of the anti-inflammatory effects of PPARs are achieved by this process [27]. The transcriptional activity of PPARs can also be modulated by post-translational modifications, such as phosphorylation, ubiquitination, and small ubiquitin-related modifiers (SUMOylation) [28]. SUMOylation functionally reduces nuclear receptor activity through transcriptional repression. A notable example of such a mechanism is the ligand-regulation of SUMO-1 to the LBD of PPARγ, resulting in the anti-inflammatory trans-repression in macrophages and inhibition of N-CoR degradation [29].

### 2.2. Liver X Receptor

The two LXR isotypes, (NR1H3) α and (NR1H2) β are activated by a specific class of oxidized derivatives of cholesterol, namely 22(R)-hydroxycholesterol [30], and 24,25(S)-epoxycholesterol [31]. LXRα is significantly expressed in metabolically active tissues, such as the liver. Its expression is low in adipose tissue, intestine, and kidney, whereas LXRβ is ubiquitously expressed [32]. The LXR isotypes share about 78% amino acid sequence identity and have the same modular architecture as PPARs. In the non-induced states, LXR is associated with corepressors, N-CoR, and the silent mediator of retinoic acid receptor and thyroid receptor (SMRT). This association causes the chromatin to become compact with non-acetylated histones due to the action of several histone deacetylases [30]. Upon binding of oxysterols and other RXR ligands to the LXR/RXR heterodimer complex, a conformational change leads to the dissociation of the corepressors, exposing binding sites for coactivators, such as the p160 family of coactivators [30], leading to LXR-dependent gene expression.

LXRs are central modulators of sterol regulatory-element-binding proteins (SREBPs) expression, which are master-regulators of de novo lipogenesis (DNL) and cholesterol synthesis [32]. In particular, SREBP-1c regulate the transcription of genes encoding fatty acid synthase (FASN), acetyl-CoA carboxylase (ACC), stearoyl-CoA desaturase 1 (SCD-1), and glycerol-3-phosphate acyltransferase [33]. The LXR-selective agonist T0901317 binds to LXR, which activates SREBP-1c gene transcription that subsequently increases the expression of its lipogenic target genes FASN and SCD-1 [33]. Synthetic LXR agonists have been shown to be beneficial in driving anti-atherogenic processes through increasing tissue cholesterol efflux and reverse cholesterol trafficking through HDL [34]. However, they may also have a deleterious effect by promoting hepatic steatosis and hypertriglyceridemia. Genetic studies have identified hepatic LXRα as the predominant subtype required for agonist-induced lipogenesis and the formation of the steatotic liver [35]. Surprisingly, LXRα/β double KO in mice led to increased DNL in adipose tissues with a marked reduction in hepatic FA synthesis and cholesterol metabolism [36], revealing the opposite role of LXRs in the regulation of DNL in these two metabolic tissues.

LXRs are also well-specialized in cholesterol metabolism and transport by activating reverse cholesterol transport through their target genes, ATP-binding cassette transporter-A1 (ABCA1), and -G1 (ABCG1) [37]. LXRα-binding site was identified in the promoter of the rodent *Cyp7a1* gene, but not in the human gene, encoding cholesterol 7α-hydroxylase, the first rate-limiting enzyme in the pathway converting cholesterol to bile acids [30]. In rodents, retinoic acid promotes cyp7a1 activity and the consequent synthesis of cholic acid by binding the LXRE in the promoter region of Cyp7a1 and inducing its expression. In contrast with that, no LXRE has been found in the human Cyp7a1 promoter, and LXR activation in humans results in the repression of Cyp7a1 [38]. Therefore, LXR-mediated effects observed in rodent models cannot be translated to humans. This assertion was corroborated in a study conducted by Peet et al. where they indicated that LXRα^−/−^ mice fed on a diet containing cholesterol fails to activate transcription of genes encoding *Cyp7a1* in contradiction to LXRα^+/+^ mice, resulting in a rapid accumulation of cholesteryl esters in the liver [39].

## 3. Transcriptional Regulation of Adipogenesis: Interplay between C/EBPs, PPARs and SREBPs

Adipocytes play a central role in maintaining lipid homeostasis and energy balance in vertebrates by storing TAG or releasing FA in response to energy demand. The mechanism behind adipocyte development is an important and fundamental biological process with crucial implications for health and disease. The path to becoming an adipocyte involves two processes. The first step involves the generation of adipocyte progenitors from multipotent mesenchymal stem cells, while the second step involves the terminal differentiation of the adipocyte progenitors into mature and functional adipocytes. Adipose tissue is a highly heterogeneous organ, with mature adipocytes primarily constituting most cells. In addition to the adipocytes, fat tissue also contains other stromal-vascular cells, such as fibroblasts, smooth muscle cells, pericytes, immune cells, and endothelial cells [40].

There are two subtypes of adipose tissues, the white adipose tissue (WAT) and the brown adipose tissue (BAT). The latter is endowed with mitochondria, necessary for the production of large amounts of heat via the actions of uncoupling protein-1 (UCP-1) [41], making BAT indispensable in the thermoregulatory function of non-shivering heat production in cold-adapted mammals [42]. The overall function of the WAT is to control whole-body energy homeostasis through metabolic and endocrine activities. Any dysregulation in the maturation of adipocytes and adipogenic differentiation processes, often as a result of impaired nuclear receptors signaling, may lead to adipocyte hypertrophy and dysfunctional adipocytes and eventually to obesity and metabolic syndrome.

**Figure 2:**
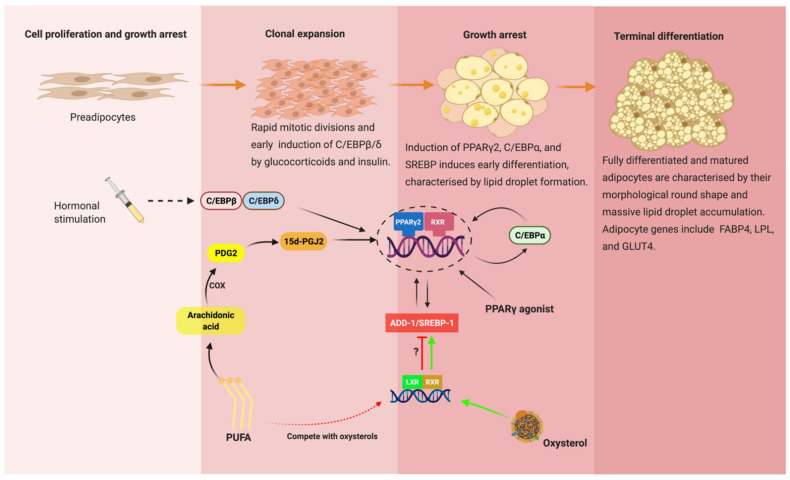
Transcriptional regulation of adipogenesis: Effective adipogenesis depends on the interdependency of several transcription factors. The treatment of preadipocytes with hormonal stimulants, such as glucocorticoids, cAMP, and insulin, transiently induces C/EBPβ and δ early in adipogenesis. Together, C/EBPβ and δ induce PPARγ2. C/EBPα, another member of the C/EBP family of transcription factors, is induced later on during adipogenesis by PPARγ2, and in turn, C/EBPα sustains the high levels of PPARγ2. The expression of ADD-1/SREBP-1, which PPARγ2 enhances, is necessary to induce target genes involved in the fatty acid synthesis, leading to the accumulation of lipid droplets, a characteristic of fully matured adipocytes. PUFA control adipogenesis via its endogenous derivative 15d-PGJ2. PUFA may also impair the induction of SREBP1 by competing with oxysterols, a potent LXR ligand. Abbreviations: ADD-1: adipocyte differentiation and determinant factor, C/EBP: CCAAT enhancer-binding protein, cAMP: cyclic AMP, PUFA: polyunsaturated fatty acid, PPAR: peroxisome proliferator-activated receptor, PDG2: prostaglandin G2, SREBP-1: sterol regulatory element-binding protein 1, 15d-PGJ2:15-deoxy-Δ12,14-prostaglandin J2. Created with BioRender.com, accessed on 16 March 2021.

In vitro models like Simpson–Golabi–Behmel syndrome (SGBS) and unipotent 3T3-L1 cells are excellent and reliable models to study the mechanism of adipocyte differentiation in humans and mice, respectively [43,44]. In culture, adipogenesis is achieved by using a defined adipogenic cocktail (insulin, triiodothyronine, cortisol, and PPARγ agonist) [43,45]. Differentiation can be enhanced by inducing agents, such as dexamethasone, used to stimulate the glucocorticoid receptor pathway, and guanyl cyclase inhibitors, such as 3-isobutyl-1-methylxanthine to stimulate the cAMP-dependent protein kinase pathway [46]. The exposure of preadipocytes to the adipogenic cocktail results in a specific and sequential change in gene expression profile that finally defines adipocyte differentiation. The major chronological event of adipogenesis is post-confluent mitosis, growth arrest, and lipid accumulation (Figure 2). After growth arrest, cells are committed to becoming adipocytes and start to express lipogenic genes as well as other adipocyte modulating proteins [46]. The regulation of adipogenic genes occurs at the transcriptional level. A group of interdependent transcription factors that orchestrate adipogenesis includes PPARγ [47], (C/EBPs) α, β, and δ [48,49], and adipocyte determination and differentiation dependent-factor 1/sterol regulatory element-binding protein 1 (ADD1/SREBP-1) (Figure 2) [48]. Collectively, they regulate the expression of mature adipocyte-specific genes, namely FABPs, LPL, CD36, GLUT4, adiponectin, and leptin (reviewed in [50]).

PPARγ is considered as the master regulator of the whole adipogenic machinery. The hormone glucocorticoids and insulin stimulants induce the early expression of C/EBPβ and C/EBPδ that together induces the expression of PPARγ early in the differentiation process [50]. In a classic positive feedback mechanism, PPARγ expression leads to the activation of C/EBPα later in the differentiation process. C/EBPα functionally synergizes with PPARγ and is involved in maintaining high levels of PPARγ expression [51]. Therefore, these two factors cooperate by mutually inducing each other and jointly activate common target genes [52]. Consistent with PPARγ being the master regulator of adipogenesis, administration of PPARγ agonist to cultured murine G8 myoblast caused accumulation of lipids and the expression of adipogenic genes [53]. Furthermore, insulin-sensitizing drugs, such as TZD, were shown to be potent and effective in stimulating adipogenesis in in vivo lineage marking (R26R^RFP^) and BrdU-treated mice [54]. Interestingly, a few sets of adipogenic genes are repressed by TZD, including the PPARγ gene itself [52,55], in matured adipocytes. The antagonistic effect of TZD on selected genes in matured adipocytes remained elusive.

Although not robustly as effective as PPARγ and C/EBPs, the regulation of adipogenesis by ADD1/SREBP-1 is possible by directly inducing PPARγ expression via E-box motifs present in the PPARγ promoter [56]. Under culture conditions favorable for adipogenic differentiation, the 3T3-L1 preadipocyte cell line expressing the ADD1 showed a marked increase in adipogenesis by enhancing the transcriptional activation of PPARγ/RXRα compared to the dominant-negative ADD1 [56]. Similarly, ectopic expression of ADD-1/SREBP-1 (rodent and human homologs) in 3T3-L1 and HepG2 cells, respectively, induced endogenous PPARγ mRNA levels [11], further reaffirming the interdependency of PPARγ and the activities of ADD-1/SREBP-1 during adipogenesis. When the transcriptional factors for healthy adipogenesis are perturbed, this can cause obesity, T2DM, and NAFLD. The function of PPARγ and its transcriptional partners in adipogenesis and their inseparable relationship with obesity and T2DM has become the target of research for future potential drug discoveries.

## 4. The Interdependent Role of Metabolic Lipases and Nuclear Receptors (PPARs and LXR)

Free fatty acids, a product from the cytoplasmic TAG degradation through lipolysis by intracellular metabolic lipases, are necessary fuel for cellular catabolism as well as potent signaling molecules that serve as ligands for the nuclear receptors. In turn, activated nuclear receptors modulate the transcription of lipase genes to direct fuel molecules to the appropriate metabolic pathways, suggesting an interdependent role of these metabolites (Figure 1A).

### 4.1. Adipose Triglyceride Lipase (ATGL)

ATGL or patatin-like phospholipase domain-containing protein A2 (PNPLA2) was first described in 2004 by Zimmermann et al. [57]. In this study, hormone-sensitive lipase (HSL) null mice were neither obese nor cold-sensitive and failed to completely abolish lipolysis [58]. Further investigations, prompted by those unexpected results, led to the identification of ATGL as the main intracellular triglyceride lipase. ATGL is highly expressed in adipose tissues and to a lesser extent in the liver, heart, muscle, intestine, and pancreatic β cells [57], suggesting the wider role of the enzyme in energy homeostasis. Enzymatically, ATGL executes the first and committing step in TAG hydrolysis, in which TAG is catabolized to diacylglycerol (DAG) and FFAs. The active site of ATGL contains an unusual Ser–Asp catalytic dyad within the patatin domain [59]. Its C-terminus encompasses a hydrophobic region required for binding to lipid droplets (LDs) [60], where it initiates lipolysis [59]. The stereospecificity of ATGL in vitro showed a preference for the sn-1 and sn-2 positions of TAG and very weak activity against DAG, and no activity against cholesterol and retinyl ester bonds [60].

It is worth mentioning that the activity of ATGL is largely enhanced by a coactivator protein, comparative gene identification-58 (CGI-58). Although CGI-58 does not have a lipase or esterase activity per se, its N-terminus lipophilic tryptophan-rich region is essential for the localization and stimulation of ATGL [61]. Recently, PNPLA3, the closest homolog to ATGL/PNPLA2, was also found to localize on LDs. It was suggested that PNPLA3 might directly affect LD size since it was shown to possess a TAG hydrolase activity [62]. However, the accumulation of PNPLA3 is limited due to ubiquitylation and proteasomal degradation. Its variant, I148M, can circumvent this ubiquitylation machinery and, therefore, accumulates on the LDs to a greater extent [63]. Interestingly, another way for PNPLA3 to affect the size of LDs may be through its interaction with CG1-58 and ATGL. In CGI-58 KO mice, PNPLA3 did not adequately accumulate on LDs compared to WT mice, while the overexpression of PNPLA3 (I148M) caused a significant increase in hepatic TAG levels in WT compared to CGI-58 KO mice [64]. This suggests that PNPLA3 I148M may sequester CGI-58, restricting its access to ATGL, thus impairing lipolysis and favoring LD accumulation [64].

The crucial role of ATGL in lipid metabolism became apparent when ATGL knockout mice showed an increased fat mass and TAG deposition in the heart, causing cardiac dysfunction and premature death [65]. On the other hand, the ablation of ATGL in HFD fed animals proved to be beneficial in the amelioration of obesity and associated metabolic syndrome and NAFLD [66,67,68]. The extent of ATGL expression does not always correlate with cellular lipase activity. A classic example was a study conducted by Kralisch et al. in which, despite the reduction of *ATGL* gene levels in adipocytes upon isoproterenol and tumor necrosis factor (TNF) α treatment, the ATGL lipolytic activity remained unchanged [69]. This may suggest that cellular lipase mRNA levels alone are not sufficient as indicators for enzyme activities, and therefore, post-translational modifications and enzymatic activity of ATGL must be considered in experimental investigations.

In adipose tissue, ATGL is regulated by PPARγ at the late phase of adipogenesis [70]. The expression of ATGL in preadipocytes is downregulated by the basal transcription factor Sp1. The extent of this downregulation is based on the interactions between Sp1 and PPARγ. In mature adipocytes, PPARγ abolishes the effect of Sp1 transcriptional repression activity at the ATGL promoter and, therefore, upregulates the expression of ATGL mRNA [70]. Insulin is known to inhibit ATGL activity. Therefore, prolonged and uncontrolled WAT lipolysis, often exacerbated by IR, produces an excessive flux of FFAs to the liver [66]. The perpetuation of this insult kick-starts developing NAFLD [66]. Targeting ATGL in adipose tissue, therefore, seems to be an attractive approach to reduce plasma FFAs concentration and its concomitant lipotoxic impact on the liver. Schweiger et al. showed that pharmacological inhibition of ATGL using atglistatin effectively reduced adipose tissue lipolysis, weight gain, IR, and NAFLD in HFD-fed mice compared to untreated diet-matched mice [66].

Nonetheless, the FFA released, as a result of lipolysis, are important lipid-sensing ligands for the NR1 and NR2 subfamily of nuclear receptors. The PPARα agonist, fibrate ameliorates steatotic and inflammatory conditions in livers of ATGL knockout mice fed with a methionine-choline-deficient diet and lipopolysaccharide (LPS) as a model of hepatic inflammation relative to untreated mice [71]. ATGL knockdown mice fed on an HFD showed downregulation of hepatic mitochondrial oxidation [72]. In the liver, the liberation of FFAs mediated by ATGL activates PPARα, therefore, positively regulating mitochondrial β-oxidation.

Besides the direct effect of PPARγ modulation on ATGL, nuclear receptors may also exert an indirect regulatory role. For example, G0/G1 Switch Gene 2 (G0S2) is a selective inhibitor of ATGL and a direct target of LXRα since LXRα knockout mice exhibited decreased hepatic G0S2 expression [73]. The G0S2 expression is responsive to adipose-derived FAs flux to the liver and modulates hepatic substrate utilization through lowering available FAs while increasing the rates of glycogen breakdown [73]. LXRα is known to be a mediator of the hepatic response to fasting and agonist-induced hepatic TAG accumulation [74], possibly via the LXRα-G0S2 axis [73]. This suggests a reasonable insight into a new mechanism by which LXRα fuels liver steatosis independent of its DNL and reverse cholesterol trafficking function.

### 4.2. Hormone-Sensitive Lipase (HSL)

HSL is an intracellular neutral lipase that exhibits broad specificity for TAG, DAG, cholesterol esters, and retinyl esters. HSL was previously described as cholesteryl ester hydrolase (CEH). The relative hydrolase activity of HSL is 11-fold higher for DAG than TAG [75]. Hence DAG is the preferred substrate. The phosphorylation of HSL by PKA (Ser-552, 649, and 650), MAPK (Ser-554), and ERK (Ser-58971) influence its activity and cellular localization [76]. These post-translational modifications facilitate the transfer of HSL to the lipid droplets [76] to orchestrate hydrolysis. Another putative mechanism by which HSL modulates adipose tissue metabolism is by providing intrinsic ligands for PPARγ. Festuccia et al. and Liu et al. demonstrated that rosiglitazone directly stimulates HSL mRNA expression levels to approximately the same extent as ATGL and adipose fatty acid-binding protein 4 (FABP4) mRNA, a direct PPRE-containing target of PPARγ [77,78]. Therefore, HSL may be associated with rosiglitazone-mediated FFA release in adipose tissue. Defects in the HSL gene have been associated with various metabolic disorders, such as fatty liver [79], hyperglycemia [80], and hyperinsulinemia [81]. However, the association of HSL with developing hepatic steatosis has been demonstrated to be as a result of dysfunctional HSL in the adipose tissue rather than in the liver, since AT-specific, but not liver-specific HSL KO induces the phenotype of global HSL KO in mice [79]. This supports the notion that, in defining the mechanisms of hepatic steatosis in the context of HSL, it is paramount to target the adipose tissue rather than the liver.

In a human clinical study, HSL activity was shown to be significantly lower in obese patients when compared to healthy controls, whereas the HSL mRNA levels remained unchanged [82], further suggesting that the gene expression of HSL does not correlate to its activity.

### 4.3. Monoglyceride Lipase (MGL)

MGL is a serine hydrolase (rate-limiting enzyme) that plays a crucial role in the hydrolysis of monoglyceride (MG) derived from phospholipids and TAG into glycerol and FFAs. The pharmacological and genetic inhibition of MGL in mice led to a significant ectopic accumulation of MGs in tissues [83]. MGL is expressed in the liver, adipose tissues, intestines, and brain (reviewed in [84]). MGL is associated or localized on lipid droplets and plasma membranes. MGL is rather an interesting enzyme because it acts as a bridge between organ-specific nutrient metabolism to central and peripheral endocannabinoid and eicosanoid systems (reviewed in [84]). MGL hydrolyzes 2-arachidonoyl glycerol (2-AG), an endogenous signaling lipid that activates the cannabinoid receptors (CB1R and CB2R) into arachidonic acid. The arachidonic acid generated serves as a lipid precursor for the eicosanoid signaling pathway, providing useful ligands for PPARs thereof. Therefore, MGL represents an important substrate provider in multiple organs for several intersecting biological pathways ranging from FA metabolism [59] to inflammation [85]. Despite its crucial role in lipid metabolism, very little is known about the regulation of MGL. Lack of MGL impairs lipolysis and is associated with increased MG levels in adipose and non-adipose tissues [59]. In HFD-fed murine models, MGL ablation is protective in developing glucose intolerance and IR, although reduced MGL activity was partially reverted by HSL [86].

Microarray analyses revealed that MGL is regulated at the transcriptional level by PPARα in mouse livers [87]. In line with that, treatment of HFD-fed mice with a PPARα agonist (Wy14643) reduced hepatic lipid content due to the upregulation of MGL compared to untreated HFD fed mice [87]. The absence of MGL protected mice from hepatic steatosis by promoting β-oxidation and lipogenesis in WAT while repressing intestinal fat absorption and crucial lipogenic and inflammatory genes in the liver. Intestinal fat malabsorption and increased lipogenesis in WAT favor AT fat storage and prevent ectopic fat accumulation in the liver [88]. The intestinal fat malabsorption and increase lipogenesis in WAT favor adipose tissue fat storage and prevent ectopic fat accumulation in the liver. MGL ablation ameliorates LPS-induced inflammation, while global or whole-body genetic and pharmacological inhibition of MGL protected against inflammation and liver lesions provoked by ischemia/reperfusion injury [85].

### 4.4. Lipoprotein Lipase (LPL)

Lipoprotein lipase (LPL) is abundantly expressed in the capillaries of adipose tissue, muscle, skeletal muscles, heart, mammary gland, and at lower levels in the liver, adrenal, and brain [35]. The LPL acts as a gatekeeper for the entry of FAs into tissues and controls the systemic lipid partitioning necessary for energy homeostasis of the body. Upon LPL-mediated partial hydrolysis of the TAG core of chylomicrons and VLDL, the main carriers of lipids in the bloodstream, FAs are taken up by the tissue and are either re-esterified and stored in adipose tissue, metabolized as an energy source in peripheral tissues, or channeled in lipid synthesis pathways. LPL activity is also essential for the processing of TAG-rich lipoproteins into HDL [89].

The activity of LPL, like other lipases, is tightly regulated by nuclear receptors. The abnormal expression of the LPL gene is linked to various metabolic diseases [90]. The knockdown of LPL effectively downregulates the expression of C/EBPα and PPARγ in human adipose stem cells in contrast to wild-type cells [91]. The detailed action of the mechanism of the LPL-C/EBPα-PPARγ axis regulating adipogenic differentiation is not entirely clear. The LPL transcriptional regulation became evident when rats fed an HFD with COOH, a non-TZD PPARγ agonist, increased LPL mRNA activity in the subcutaneous fat compared to the non-treated HFD fed rats [92]. The activity of LPL is also modulated by altering the production of proteins that assist in LPL enzymatic activity. These groups of proteins include APO-CII, APO-CIII, APO-AV, and angiopoietin-like protein 3 and 4. APO-CIII, a potent inhibitor of LPL activity, is downregulated by PPARγ [93] and PPARα [94,95]. The possible mechanism of action behind the downregulation of APO-CIII by PPARα may involve Rev-erbα [96] and HNF4α [97]. In contrast, PPARα agonist upregulate hepatic expression and plasma levels of APO-AV [98].

In the liver, LPL promotes the uptake of HDL cholesterol, hence, facilitates reverse cholesterol transport. Mice fed a high cholesterol diet or treated with LXR agonist T0901317 upregulates LPL mRNA expression in the liver and macrophages, but not in other tissues compared to chow diet-fed or untreated mice, suggesting a tissue-specificity of the LXR regulation of the LPL gene [90]. The likely physiological explanation of tissue-specific regulation of the LPL gene by LXR may be attributed to the role LXR plays in maintaining whole-body cholesterol homeostasis in the liver. In vivo and in vitro respective treatment of adult rats and hepatocytes with fibrates (fenofibrate, clofibrate, or gemfibrozil) increases the hepatic LPL expression compared to untreated conditions [15]. Although PPAR agonists influence lipid trafficking by altering the gene expression of LPL in the liver and adipose tissue, TZD induces LPL mRNA and activity levels in adipose tissues but not hepatic LPL expression [15]. This suggests that different agonists have different tissue specificity.

## 5. Physiological and Pharmacological Ligands for PPAR and LXR

FAs are important biomolecules for many cellular processes; therefore, it is reasonable to assume that their levels must be tightly regulated since dysregulation of these biomolecules often results in metabolic syndrome. Organisms possess lipid sensing nuclear receptors for precisely controlling FAs level via the regulation of metabolic lipases and other processes. In essence, FAs modulate the transcription of genes involved in their metabolism. The three PPAR isotypes can bind to FAs. However, saturated fatty acids (SFA) are poor ligands, whereas the easily oxidized polyunsaturated fatty acids (PUFA) have a greater binding affinity to PPARs [99,100].

Linolenic acid regulates adipogenesis and represses many transcription factors associated with lipid and carbohydrate metabolism [101,102]. As an example, transgenic mice carrying the fat-1 gene from Caenorhabditis elegans can synthesize omega-3 PUFAs and, therefore, have higher tissue levels of omega-3 PUFAs relative to wild-type mice. Omega-3 PUFAs overload reduces white adipocyte progenitor (WAP) population and suppresses their differentiation in Fat-1 mice compared to wild-type mice [103]. The reduction of WAP by omega-3 PUFA is possibly achieved through the partial suppression of platelet-derived growth factor receptor (PDGFR)α, which regulates WAP proliferation through the phosphatidylinositol 3-kinase/AKT serine/threonine kinase 2 (PI3K/AKT2) pathways [103,104]. In line with this, expression of PDGFRα in Fat-1-stroma vascular fraction was low relative to wild-type- stroma vascular fraction [103]. Similarly, treatment of 3T3-L1 cells with linoleic acid resulted in the repression of SCD-1 [105], a rate-limiting enzyme in DNL and transcriptional target of SREBP-1c. The suppressive effect of PUFA on SREBP-1c expression [106] may be due to PUFA competing with LXR ligands during activation of the ligand-binding domain of LXR, thus inhibiting the binding of LXR/RXR heterodimers to the LXREs in the SREBP-1c promoter [107].

### 5.1. Endogenous and Synthetic Ligands for PPARα

PPARα activation through FAs mainly occurs during starvation or energy deprivation and leads to the upregulation of intracellular energy metabolism, ultimately inducing ATP production from oxidative phosphorylation. During starvation, the rate of lipolysis increases, and the released FFAs activate PPARα and the stimulation of the β-oxidative enzymes, such as carnitine palmitoyltransferase 1A and 2 (CPT1A and 2) [108]. Eicosanoid derivatives, such as chemoattractant leukotriene (LTB4), 8S-hydroxyeicosatetraeinoic acid (8S-HETE), and the murine 8- lipoxygenase (8-LOX) are endogenous PPARα agonists [109,110,111].

Fibrates, a group of synthetic ligands that induce peroxisome proliferation and FAO in rodents was first demonstrated in vitro to activate PPARα. Relative to placebo, diabetic patients receiving 0.2 mg/day pemafibrate (a selective PPARα agonist) for 24 weeks showed marked reduced fasting serum TAG, non-HDL, and remnant lipoprotein cholesterol, ApoB100, ApoB48, ApoCIII levels as well as increased HDL-cholesterol and ApoA-I levels [112]. Although selective PPAR agonists positively control lipid and carbohydrate metabolism, PPAR dual agonists are optimal in this regard. PPARα/γ dual agonist, also known as glitazars, works as both fibrates and TZD and effectively alleviates T2DM [113]. The novel 1,2,4-oxadiazole based trans-acrylic acid derivatives, dual PPARα/γ agonists significantly reduce plasma glucose and cholesterol levels in severely obese rats [113]. However, so far, glitazars have always failed in clinical trials due to increased cardiovascular diseases [114].

### 5.2. Endogenous and Synthetic Ligands for PPAR-β/δ

PPAR-β/δ binds to several PUFA in a pattern similar to PPARα [115]. Prostacyclin (PGI_2_) is a major prostaglandin derived from arachidonic acid through LOX or cyclooxygenases (COX) activity and can activate PPAR-β/δ in vivo [116], suggesting that a novel signaling mechanism for this abundant eicosanoid is operative in certain systems. In addition to PGI_2_, other metabolite derivatives, such as 9-HODE, 13-S HODE, 12-HETE and 15-HETE, can also activate PPAR-β/δ [117] (Figure 1A). Interestingly, 13-S HODE inhibits PPAR-β/δ in the colon epithelial cells [118].

L-165043 was the first PPAR-β/δ agonist discovered at Merck but proved to be ineffective in reducing glucose and TAG in leptin receptor-deficient mice [119]. This led to the discovery of GW501516, a well-known effective PPAR-β/δ agonist. GW501516 increase FA oxidation in the skeletal muscle, reduce fat gain in HFD fed mice, and promote increased glucose tolerance and insulin sensitivity in the ob/ob mouse model of obesity and IR [120]. Although GW501516 demonstrated a favorable pharmacological profile, it was later withdrawn as a drug due to cancer promotion in preclinical animal testing [121]. GW0742, which was developed together with GW501516, is a highly selective PPAR-β/δ agonist commercially available for non-human research purposes [120]. A relatively novel selective PPARβ/δ agonist, MBX-8025/RWJ800025 (seladelpar), has been shown to improve insulin sensitivity and reverses dyslipidemia and hepatic storage of lipotoxic lipids and improve NASH pathology in atherogenic diet-fed obese diabetic mice [122]. To confirm the efficacy of seladelpar observed in the above preclinical models in NASH human patients, a 52-week multicenter, double-blind, randomized phase 2 clinical trial of Seladelpar in NASH cohorts was conducted. Although seladelpar treatment reduced key liver enzyme levels and lead to modest NASH and fibrosis improvements, these were not statistically significant compared to the placebo group [123]. Moreover, the discovery that PPAR-β/δ promotes psoriasis is a major caveat for developing new PPAR-β/δ agonists, which will require an excellent tissue selectivity [124].

### 5.3. Endogenous and Synthetic Ligands for PPARγ

Endogenous FAs and their derivatives are putative PPARγ ligands [109]. Kliewer et al. showed that 15d-PGJ2 binds directly to PPARγ and promotes the differentiation of 3T3-L1 and 3T3-F422A preadipocytes to mature adipocytes [125]. On the other hand, Reginato et al. reported that another prostaglandin, PGF2α, blocks adipogenesis by activating mitogen-activated protein kinase (MAPK), resulting in inhibitory phosphorylation of PPARγ [126]. These seemingly contradictory roles of prostaglandins may be somewhat a regulatory function concerning the prevention of hypertrophy of the adipose tissues.

TZD (troglitazone, rosiglitazone, and pioglitazone) are insulin-sensitizing compounds used to treat diabetes [127]. PPARγ activation by TZD causes a decrease in FFA levels and increased lipid storage in adipose tissues. Activation of PPARγ by TZD affect fat cell distribution and improves insulin sensitivity in troglitazone treated T2DM patient [128]. A head-to-head clinical study between pioglitazone and rosiglitazone showed that, although both agonists reduced IR and improve glycemic control in patients with T2DM, pioglitazone was associated with significant improvement of TAG, HDL cholesterol, and LDL particle concentration compared to rosiglitazone [129]. These differences in the outcome of the PPARγ agonist on metabolic processes need to be explored further, nonetheless. Furthermore, many randomized controlled clinical trials have also reported that both rosiglitazone and pioglitazone improve NAFLD-related hepatic steatosis and, in the case of pioglitazone, also hepatic inflammation and, to a lesser extent, fibrosis [20,21,22,23]. In a multicenter, randomized, double-blind, parallel-group, placebo-controlled study involving 173 patients, lobeglitazone (0.5 mg) significantly improves IR, FFA, TAG, HDL cholesterol, small dense VLDL cholesterol, and ApoB/CIII in T2DM patients [130].

Although TZDs have been proven to be highly efficient insulin-sensitizers, their use has been challenged in clinical practice because of their side effects, such as weight gain, fluid retention, and edema, which may explain their cardiac side effects [131], whereas bone fractures increased [132]. Selective PPARγ modulators (SPPARMs) have been shown to improve glucose homeostasis and insulin sensitivity with reduced side effects due to partial PPARγ agonism based on selective receptor-cofactor interactions and target gene regulation [133]. N-acetylfarnesylcysteine is an example of a SPPARM compound with both full and partial agonists depending on the investigated PPARγ target gene [134].

### 5.4. Endogenous and Synthetic Ligands for LXR

Oxysterols, namely 24S-hydroxycholesterol, 25-hydroxycholesterol, and 27-hydroxycholesterol, are important endogenous ligands for the activation of LXRα and LXRβ in vitro [30] and regulate the expression of genes involved in cholesterol and FA metabolism in vivo [39]. However, whether these oxysterols truly activate LXR under in vivo conditions has proven difficult to establish thus far. Therefore, it is reasonable not to exclude the role unmodified cholesterol may have on LXR activation since cholesterol feeding induces LXR target genes by increasing oxysterol levels [39].

FAs may compete with the oxysterols and thus, inhibit the LXR activation [135]. However, the extent of the inhibition is predicated on the degree of unsaturation of the FAs since PUFAs are more potent inhibitors of oxysterol binding in contrast to monounsaturated fatty acid [136]. PUFA (20:4, n6) suppressed LXRα activity but did not affect LXRβ [136]. Although saturated medium-chain fatty acids activate LXRα, the ligand-binding affinities of saturated medium-chain fatty acids are in the low nanomolar concentration range [135]. It is likely that PUFA-inhibition of LXR mediated the suppression of SREBP-1c at both the gene and protein level in the animal liver [137].

LXR activation is both beneficial and deleterious to numerous metabolic processes. Combined agonists, T0901317 and fenofibrate improved IR and glucose tolerance and worsened the hepatic steatosis in HFD fed mice compared to untreated mice [138]. GW3965, a synthetic LXR agonist, ameliorates diet-induced obesity, IR, and glucose tolerance in mice [139]. Gene expression analyses in LXR agonist-treated mice showed coordinate regulation of genes involved in glucose metabolism in liver and adipose tissue, particularly induction of glucokinase and downregulation of peroxisome proliferator-activated receptor γ coactivator-1α (PGC-1α) in the liver, and induction of insulin-sensitive glucose transporter 4 (GLUT4) in the adipose tissue [139].

## 6. PPARs and LXRs in Adipose Tissue Metabolism

The liver and adipose tissue play a significant role in maintaining metabolic flexibility. Although they are robust in regulating whole-body energy homeostasis, they are evolutionarily not built to cope with a chronic nutrient surplus seen in the obese state. Within the liver, glucose and lipids metabolism is intricately linked and tightly regulated by nuclear receptors. In this section, we will elaborate on how dysregulation of adipose tissue metabolism during sustained metabolic stress (obesity), lipid fluxes, IR, and inflammation lead to developing hepatic steatosis and inflammation in the liver.

Adipose tissue increases in size in two distinct ways: hypertrophy, increase in the size of existing adipocytes, and hyperplasia, the generation of new adipocytes from resident preadipocytes [140]. One putative link between obesity and global IR is how adipocytes expand their fat storage ability, an ability orchestrated by PPARγ. Troglitazone treatment normalizes hyperglycemia, marked hyperinsulinemia, and plasma TAG reduction in obese Zucker rats [141]. The plasma TAG reduction was the result of hyperplastic adipocytes sequestrating FFA. Hence, PPARγ activation favors efficient fat accumulation in the subcutaneous tissues. Obese individuals with enlarged abdominal adipocytes were more susceptible to hyperinsulinemia and glucose-intolerant than individuals with smaller adipocytes [142,143]. Consistent with the above studies, individuals with hypertrophic relative to hyperplastic obesity were more likely to develop diabetes and IR [144].

PPARγ may also be involved in regulating FA metabolism through the modulation of FFA transporters, lipogenic, and lipolytic genes that ensure trapping or uptake of FFA. TZD upregulates the expression of CD36 and fatty acid transport protein on the surface of 3T3-L1 adipocytes [145]. Systematic and coordinated regulation of circulating FFA is necessary to ensure that they are stored appropriately in adipose tissue. It prevents “ectopic” storage in other sites, such as the liver and skeletal muscles, where they can induce “lipotoxicity” and the concomitant injury to these tissues.

In addition to adipogenesis, PPARγ regulates insulin sensitivity in adipocytes and at the systemic level. PPARγ activation affects the insulin signaling pathway through the direct modulatory effect on the expression or phosphorylation of specific insulin signaling apparatus [146]. The binding of insulin to its receptor evokes a signaling cascade that involves phosphorylation of the insulin receptor substrate (IRS) proteins and activation of phosphatidylinositol-3-kinase (PI3K), Akt, and other downstream kinases, which drive glucose uptake and other biological processes. Treatment with troglitazone increased insulin-stimulated IRS-1-associated PI-3-kinase and Akt activity in skeletal muscle biopsies from T2DM patients [146]. Another potential mechanism of action of PPARγ on adipose tissue insulin sensitivity may involve the enhancement of adipose tissue FFA storage capability by stimulating lipid uptake and storage. In the hyperplasic state, the increased adipocyte differentiation induced by PPARγ significantly increases the number of small insulin-sensitive adipocytes and augments insulin-stimulated glucose, FFA, and TAG uptake from the circulation, preventing skeletal muscles and hepatic lipid overburden [147].

PPARα expression levels in WAT are low, suggesting a limited role in adipocyte differentiation and function [14]. Nevertheless, its activation elicits systemic effects on adiposity and IR in obese mouse models. The activation of PPARα by bezafibrate significantly reduced adiposity in KK mice fed an HFD compared to the chow-fed group [14]. The bezafibrate reduction of adiposity may be attributed to increased expression of genes involved in FAO in adipose tissue, which relies largely on FFA [14]. However, bezafibrate being a PPAR pan agonist, this effect may come from its PPARγ activation capacity [148]. The PPARα agonist Wy-14,643 directly enhances lipolysis in isolated adipocytes [149] and the suppression of obesity-induced inflammatory cytokines, such as TNF-α and MCP-1 in WAT [14]. This anti-inflammatory effect in the WAT suggests PPARα activation can improve IR and ameliorate obesity. As proof of this concept, treatment with PPARα selective agonists (fenofibrate, ciprofibrate, and GW9578) significantly improves hyperinsulinemia and hyperglycemia in both HFD-fed mice and genetically obese Zucker rats [150].

The ubiquitous expression of PPAR-β/δ distribution in mammalian tissues enables it to exert a powerful regulatory function on metabolism and energy homeostasis. The metabolic activities of mature adipocytes are based on the efficiency of adipogenesis. Fibroblasts and preadipocytes expressing PPAR-β/δ respond to the LCFA by transcriptional activation of CD36, adipocyte lipid-binding protein (ALBP), and PPARγ promoting terminal adipogenic differentiation [151]. In contrast to the PPAR-β/δ upregulation of CD36 during adipogenic differentiation, in mature adipocytes, the opposite effect happens, since the PPAR-β/δ agonist GW501516 decreases the expression of CD36 levels in cultured matured adipocytes, thus improving insulin response [152]. Targeted activation of PPAR-β/δ in adipose tissue causes a significant decrease in fat mass, mainly due to the downregulation of FAs transporters, activation of FAO, and energy dissipation pathways. The acute treatment of Lepr^db/db^ mice with a PPAR-β/δ agonist reduces lipid accumulation, whereas PPAR-β/δ-deficient mice challenged with HFD show reduced UCP-1 gene expression and were susceptible to obesity [153].

Although the vast majority of studies on LXR were in non-adipose tissues, recent studies have shown that LXR may have a modulatory role in adipose tissue metabolic function. In 3T3-L1 adipocytes, T0901317 markedly increases lipogenic gene expression, such as FASN, ADD1/SREBP1c, and PPARγ [154]. Against this backdrop, LXR expression in 3T3-L1 and SGBS preadipocytes was also shown to be regulated by PPARγ and C/EBPα [155,156]. Treatment of 10 non-diabetic patients (identified as having low IRS-1 and GLUT4 protein in the adipose cell) for 3 weeks with pioglitazone resulted in significant upregulation of adipose tissue LXRα mRNA expression relative to non-treated groups [157]. The role of LXR in both liver and adipose tissues may function in an opposing manner. Impaired hepatic lipogenesis in LXRαβ-knockout mice was shown to be accompanied by a reciprocal increase in adipose lipid storage by promoting adipose SREBP, PPARγ, and ChREBP lipogenic pathway activity [32]. This indicates possible crosstalk between LXR and PPARγ in adipose tissue. LXR is also a glucose sensor and can regulate glucose homeostasis and insulin sensitivity [158]. The insulin-responsive GLUT4 plays a crucial role in insulin-mediated facilitated glucose uptake into adipose tissue and muscle, and impaired expression of GLUT4 has been linked to obesity and diabetes [139]. Human and murine GLUT4 promoters harbor the functional LXRE, which, when activated by LXRα/RXR heterodimer, induces the activity of the reporter construct facilitated by the GLUT4 promoter [139].

## 7. PPARs and LXRs in Adipose Tissue Inflammation

**Figure 3:**
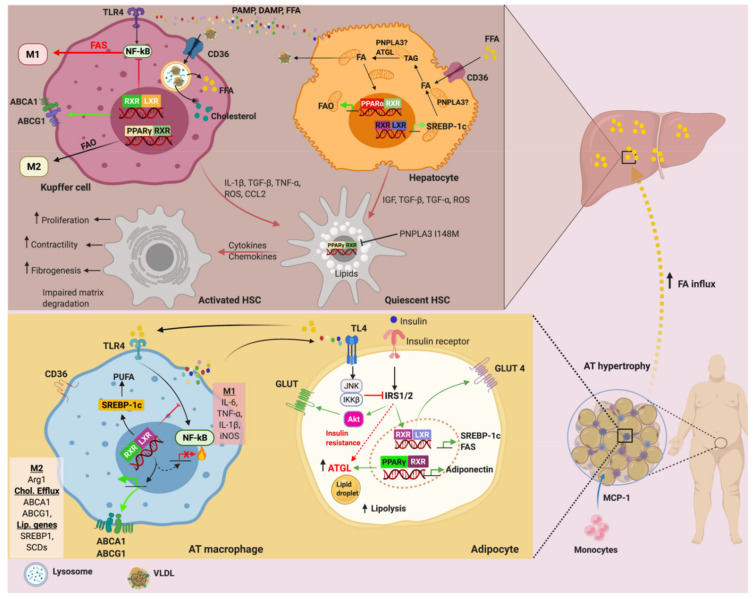
Nuclear receptors and adipose-liver axis in insulin resistance and NAFLD: FAs from hypertrophic adipocytes serve as ligands for TLR4 to induce NF-κB and subsequent proinflammatory cytokines and chemokines expressions in macrophages. The released cytokines, including TNF-α and IL-6 by macrophages, in turn, activate the TLR4 and induces IR via JNK-IκκB signaling pathways in adipocytes. In the IR state, ATGL under the control of PPARγ is upregulated to ensure rapid lipolysis. The released FFA influx the liver, where they are re-esterified into TAG. The inability to get rid of the FAs due to dysregulation of PPARα, LXR, ATGL and more recently PNPLA3 I148M lead to hepatic steatosis. In addition to its cholesterol homeostatic role, LXR can inhibit M1 activation whiles PPARγ promotes M2 activation in Kupffer cells. Activation of the Kupffer cells by the inflammatory cues from hepatocytes promotes local inflammatory milieu, further exacerbating steatosis and influence fibrogenesis. In brief, activated Kupffer cells and hepatocytes secretes IL-1β, TGF-β, TNF-α, CCL2 resulting in the activation of HSC. In addition, the variant PNPLA3 I148M results in the inhibition of PPARγ via JNK signaling pathways, thus blunting antifibrogenic action and promoting HSC activation [166]. ATGL: adipose triglyceride lipase, CCL2: C–C motif chemokine ligand 2, HSC: hepatic stellate cells, IL-6: interleukin 6, IL-1β: interleukin 1β, IκκB: inhibitor of nuclear factor κB, IR: insulin resistance, LXR: liver X receptor, JNK: c-Jun N-terminal kinases, NF-κB: nuclear factor κB, PPAR: peroxisome proliferator-activated receptor, PNPLA3: patatin-like phospholipase domain containing 3, SFA: saturated fatty acid, TLR4: toll-like receptor 4, TNF-α: tumor necrosis factor α, TGF-β: transforming growth factor β. Created with BioRender.com, accessed on 16 March 2021.

The relationship between obesity and IR involves two close yet independent mechanisms; lipotoxicity and low-grade inflammatiHans Popper Laboratory of Molecular Hepatology, Department of Internal Medicine III, Division of Gastroenterology and Hepatology, Medical University of Vienna, 1090 Vienna, Austria; emmanuel.dixon@meduniwien.ac.at (E.D.D.); alexander.nardo@gmail.com (A.D.N.); thierry.claudel@meduniwien.ac.at (T.C.) on in WAT. The former is causative for the latter. Over-nutrition can lead to adipocyte hypertrophy, lipotoxicity, and cell death. In the attempt to restore adipose tissue homeostasis under these circumstances, adipocytes releases adipokines, cytokines, and chemokines, which elicit macrophages infiltration and activation to restore normalcy as well as low-grade inflammation (Figure 3).

In the long-term, this response is maladaptive and often leads to IR. The treatment of SGBS adipocytes with macrophage conditioned medium significantly reduces glucose uptake and insulin sensitivity relative to untreated cells [159]. Nonetheless, in some instances, obesity-induced IR precedes macrophages infiltration and accumulation. In a genetically induced adipose tissue-specific IR (mTORC2-KO), the IR state caused the upregulation of MCP1 compared to wild-type and healthy mice [160]. In spite of the unclarity of the causal relationship of these events, most studies have shown that the prevention of macrophage accumulation in adipose tissue improves insulin sensitivity in various animal models of obesity [161,162].

The adipose tissue depots of healthy and obese animals contain a large population of innate and adaptive immune cells, numerically dominated by macrophages surrounding the adipocytes and the vasculature [163]. The physiological condition of adipose tissues and environmental cues (damaged cells and toxic substances) determines the extent of the inflammatory response [164]. The macrophage classical M1 phenotype, induced by LPS, IFN-γ, and GM-CSF, is characterized by high levels of proinflammatory cytokines, such as IL-1β, TNF-α, IL-6, IL-18, and IL-23 as well as reactive oxygen and nitrogen species [164]. This helps to drive antigen-specific Th1 inflammatory responses. M1 macrophages are implicated in developing IR and the aggressive form of NAFLD [162]. Alternatively, M2 macrophages are stimulated by Th2 cytokines, such as IL-4 or IL-13 and produce an anti-inflammatory response. The counteraction of M2 promotes tissue remodeling and homeostasis. However, when the lesion is persistent, the M2 macrophages assume a pro-fibrotic role and secretes pro-fibrotic factors, such as TGF-β, as often seen in the case of liver fibrosis [165].

PPARγ transcriptional signaling is required for the maturation of the anti-inflammatory M2 phenotype, wound-healing responses, phagocytosis, decreased inflammation and apoptosis, and increased lipid uptake in macrophages [167,168,169]. Studies using PPARγ-deficient macrophages, however, have shown that at least some of these effects are independent of PPARγ [168]. Szanto et al. showed that IL-4 signaling increased PPARγ activity via the interaction with STAT6 on promoters of PPARγ target genes [168]. When PPARγ was ligand-stimulated, it was not sufficient to drive the polarization of specific gene expression signature, suggesting that the STAT6 acts as a facilitator for PPARγ in the context of macrophage polarization [168]. Regardless, PPARγ ligands, such as 15d-PGJ2 and TZD improve insulin sensitivity in the adipose tissue through MCP-1 inhibition [147] and antagonization of iNOS, TNF-α, and IL-6 in response to macrophage activation [170].

In contrast to the well-established roles of PPARα and PPARγ in obesity, diabetes, and NAFLD, little is known about PPAR-β/δ despite its ubiquitous expression. The adipogenic activity of PPAR-β/δ and its involvement in inflammation raises the question of whether it is associated with hepatic steatosis and steatohepatitis. The transcriptional analysis of PPAR-β/δ-null mice showed downregulation of genes involved in lipoprotein, glucose metabolism, and upregulation of genes associated with hepatic inflammation compared to wild-type mice [171]. Bone marrow adoptive transfer of PPAR-β/δ null mice into wild-type mice diminishes the alternative M2 Kupffer cells, causing hepatic dysfunction and systemic IR [172], suggesting PPAR-β/δ involvement in the activation of alternative phenotype in Kupffer cells. PPAR-β/δ antidiabetic functions [152] seem to be intertwined with reduced inflammatory signaling. In the T2DM rat model, GW0742 was shown to reduce the proinflammatory cytokines TNF-α and MCP-1 in liver tissues, with a concomitant reduction of hepatic fat accumulation [173].

LXR regulates the inflammatory response, and this ability is dependent on changes in lipid metabolism (Figure 3). However, the mechanism behind LXR suppression of inflammation is less understood. Ito and co. demonstrated that activation of ABCA1 sterol transporter by LXR agonist alters membrane cholesterol homeostasis, which has a secondary effect on the inflammatory signal through nuclear factor-kappa B (NF-κB) inhibition [174], suggesting the unified and dual role of LXR in metabolism and inflammation. The LXR agonists can inhibit the nuclear entry of NF-κB by inhibiting the phosphorylation and ubiquitin-dependent degradation of the inhibitory κB (IκB) proteins [175]. Interestingly, without altering the binding of NF-κB to the DNA element or attenuating IκB degradation, LXR agonist can repress NF-κB activation [175] by mechanisms involving trans-repression rather than direct LXR ligand-dependent mechanism. The SUMOylation of LXR occurs in response to the LXR agonist, which causes the stabilization of repressive nuclear compounds on the promoter regions of inflammatory genes [176]. LXR agonists delayed the lysophosphatidic choline-induced degradation of IκBα in endothelial cells [175]. Additionally, the nuclear receptor corepressor (NCoR) causes basal repression of inflammatory genes [177]. However, in a study conducted by Li et al., the deletion of macrophage NCoR in mice surprisingly led to an anti-inflammatory, and insulin-sensitive phenotype, which was mechanistically traced to the derepression of LXR [177]. Therefore, it is reasonable to assume that the major effect of NCoR in macrophages is to derepress LXR, which leads to the induction of lipogenic, reverse cholesterol transport, and inflammatory pathway genes.

On the other hand, there are some instances where inflammation modulates the activity of LXR and its downstream target genes. Activation of Toll-like receptors (TLR) 3 and 4 by microbial ligands block the induction of LXR target genes in cultured macrophages and in aortic tissue in vivo [178]. This counter-regulatory action between LXR and inflammatory signaling needs further research. Collectively, these studies reasonably recapitulate the important role played by nuclear receptors in adipose tissue and macrophage immunometabolism.

## 8. PNPLA3, ATGL, PPAR and LXR in NAFLD: A Brief Update

NAFLD is one of the most common causes of chronic liver injury globally due to the increase in obesity, T2DM, and IR. NAFLD encompasses a spectrum of disorders, beginning as benign steatosis with the potential to advance to more aggressive hepatic pathologies, such as NASH, fibrosis, cirrhosis, and hepatocellular carcinoma [179,180]. It is incompletely understood why some patients with NAFLD develop the advanced form of the disease, while others do not [181]. Although both genetic and environmental factors are implied in NAFLD pathophysiology, the causal sequence of events leading to disease evolution is unknown yet.

The so-called “two-hit hypothesis” has been postulated, in which the “first hit” is defined by obesity and IR, the cues for hepatic lipid accumulation. This renders the liver more susceptible to multiple insults, the so-called “second hits”, such as proinflammatory mediators and reactive oxygen species that induce inflammation and fibrosis [182]. The leptin-deficient ob/ob mice were characterized by a sustained increase in hepatic lipid accumulation, and exposure to low doses of LPS was necessary to initiate inflammation and fibrosis [183]. This postulate has faced some criticism recently, nonetheless. In humans, multiple parallel factors that act synergistically in genetically predisposed individuals have also been implicated NAFLD development and progression. Rendering the “two-hit hypotheses” too simplistic and obsolete, which led to the proposal of a multiple parallel hits’ hypothesis [184,185,186]. Regardless, metabolic lipases and nuclear receptors are key modulators in the onset and progression of NAFLD. Therefore, understanding their role in lipid and glucose metabolism, bile acid homeostasis, inflammation, and fibrosis is fundamental to developing robust methods for diagnosis, risk identification, and therapy.

Aside from the environmental factors that influence metabolic syndrome and NAFLD, the propensity to develop NAFLD seems to differ among ethnic groups, suggesting a potential genetic role. A single nucleotide polymorphism (rs738409; C>G) in the PNPLA3 gene encoding the I148M variant is responsible for the heightened risk of the full spectrum of fatty liver disease [187,188]. Although several studies proved a causal relationship between PNPLA3 (I148M) and NAFLD development, the pathological mechanisms promoting this process have not been fully clarified yet. It was suggested that the PNPLA3 protein is involved in TAG hydrolase activity in hepatocytes [189,190] and retinyl esters in HSC [191]. Therefore, the guanine substitution results in the loss of TAG hydrolase activity and increased acyltransferase activity. Antisense oligonucleotide-mediated silencing of PNPLA3 reduces inflammation and fibrosis in PNPLA3 I148M knock-in mice model of NASH [192]. Similarly, the introduction of the PNPLA3 (I148M) into mice genes resulted in hepatic steatosis [193]. In contrast, neither knockout nor overexpression of PNPLA3 WT in mice resulted in steatosis [194,195]. These contrasting findings were inconsistent with the hypothesis that PNPLA3 causes hepatic steatosis due to a simple loss or gain of function. Li et al. showed that chronic overexpression of the PNPLA3 I148M variant in mouse hepatocytes causes hepatic steatosis [196], possibly through the PNPLA3 (I148M) evading the proteasomal degradation, thus effectively accumulating on the LDs. While on the LDs, the PNPLA3 (I148) could sequestrate CGI-58 away from ATGL, hence, impairing lipolysis [64]. The enzymatic activity of ATGL is enhanced by CGI-58. Chanarin–Dorfman syndrome, a rare recessive autosomal disorder caused by a point mutation in the human CG1-58 gene, is characterized by excessive accumulation of TAG in multiple tissues due to the inability of CGI-58 to activate ATGL [197].

The ATGL liver-specific KO in mice resulted in hepatic steatosis, albeit, compared to hepatic steatosis of obesity and diabetes, steatosis as a result of ATGL deficiency was well tolerated metabolically [66,198]. The adenovirus-mediated hepatic overexpression of HSL and/or ATGL in ob/ob mice and mice with HFD-induced obesity significantly reduces liver steatosis compared to WT [199], asserting the crucial role of intracellular TAG hydrolysis in preventing fat accumulation in the liver. The crosstalk between PPARγ and ATGL activity affects NAFLD development. Pioglitazone treatment of mice fed on HFD markedly increased hepatic expression of ATGL, HSL, and CPT-1a [200]. Pioglitazone also lowers serum insulin and hepatic TAG content and reduced hepatic steatosis compared to untreated mice [200]. Similarly, PPARα and γ deletion in AML12 cells significantly reduced the expressions of ATGL and CPT-1α [200]. The histological parameters of NASH improved in patients with impaired glucose tolerance or T2DM patients, who received 45 mg pioglitazone (once a day) for six months compared to placebo-treated patients [201]. Similar results were obtained with 30 mg daily for 24 months in patients with NASH and without T2DM [22]. Furthermore, a 12-month randomized controlled clinical trial in patients without T2DM also showed a significant improvement in the fibrosis stage with a daily dose of 30 mg pioglitazone [20]. Long-term use of pioglitazone 45 mg/day for 36 months improved NASH and fibrosis in subjects with prediabetes and T2DM [202]. A recent metanalysis confirmed that pioglitazone improves advanced fibrosis in NASH, even in patients without T2DM [203]. Importantly, pioglitazone impacts NASH development in a dose-dependent manner [204]. Therefore, clinical studies should also consider the polymorphisms known to modify pioglitazone pharmacokinetics, such as CYP2C8 [205]. Hence, far, only small clinical trials have uncovered several genetic polymorphisms in CYP2C8, LPL, and ADORA1 [206]. Therefore, glitazones are a logical approach for the treatment of NASH (reviewed in [23]) and displayed in a hierarchical network analysis the highest efficacy among drugs available [207].

PPARα is the major nuclear receptor involved in β-oxidation. Therefore, it contributes to the remarkable metabolic flexibility of the liver. Rodents on methionine-choline deficient diet developed moderate steatosis, while PPARα knockout provokes a severe NASH unaffected by its agonist (Wy14643) administration [208]. PPARα activation, in combination with PPARβ/δ agonism, has been shown to improves steatosis, inflammation, and fibrosis in preclinical models of NAFLD [209]. After statistical reanalysis, human data supported a very modest effect of elafibranor, a combined PPARα and PPAR-β/δ agonist on the histological resolution of NASH and fibrosis with an improvement of IR and serum lipid normalization [210]. However, a recent phase III clinical trial showed that elafibranor did not improve NASH leading to the discontinuation of drug development in this indication [211]. Phase-two data with the pure PPARδ agonist seladelpar in NASH were also disappointing [123]. The emerging therapeutic saroglitazar, a dual PPARα, and PPAR-α/γ agonist improve mouse liver injury. Compared to mice on chow diet with normal water and mice on western diet and sugar-supplemented water (WDSW), mice on WDSW treated with saroglitazar for 12 weeks reduce body weight, HOMA-IR, TAG, total cholesterol, and ALT with an improvement of steatosis, lobular inflammation, hepatocellular ballooning, and fibrosis stage [212]. However, given the side effects seen in all previous glitazar members, it would be surprising for saroglitazar to be exempted from toxicity problems. Adelmidrol, another PPARα, and PPAR-α/γ dual agonist reduce MMP-1, TNF-α, AST, ALT, TAG, while increasing HDL and adiponectin levels as well as improving histopathological changes in HFD-induced NASH mice [213]. Interestingly, the pan PPAR agonist lanifibranor showed encouraging data with NASH resolution without worsening of fibrosis in addition to a beneficial lipid profile with increased HDL cholesterol and reduced triglycerides [214].

As previously seen, LXR can modulate lipogenesis, cholesterol homeostasis, increase insulin sensitivity, and induce anti-inflammatory effects (Figure 3). Activation of LXR can lead to increased liver fat deposition and hypertriglyceridemia but can have a satisfactory antiatherosclerotic activity. The treatment of ApoE deficient mice with combined T0901317 and Notch receptor inhibitors (DAPT) markedly reduced atherosclerotic activity while reducing T0901317-mediated lipid accumulation in the liver [215]. The novel LXRα antagonist, ursolic acid (UA), significantly reduced LXR and SREBP1c gene expressions and their lipogenic target genes, as well as the reduction in hepatic cellular lipid content in the T0901317-induced fatty liver mouse model [216]. UA competes with T0901317, leading to the blockage of T0901317-mediated LXRα activation at the LXR ligand-binding domain [216]. The impairment of LXR activity often causes intracellular accumulation of cholesterol and the disruption of mitochondrial and endoplasmic reticulum (ER) structural integrity. This dysfunction subsequently results in mitochondrial damage and ER stress, triggering inflammation (reviewed in [217,218]). Hence, these data suggest that LXR is closely related to intrahepatic inflammation and fibrosis [176]. LXR counteracts LPS induced inflammation in macrophages [219], while LXR agonist ameliorated LPS-induced liver injury in mice fed an HFD [220]. The improvement of liver injury by the activation of LXR was reflected by the reduction in ALT and AST as well as TNFα and iNOS through the inhibition of the phosphoinositide 3-kinase (PI3K) and c-Jun N-terminal kinase (JNK) signaling pathways [220]. The novel liver-specific LXR inverse agonist SR9243 significantly attenuated hepatic inflammation and fibrosis, concurrently reducing body weight, serum glucose, and plasma lipid levels of high-cholesterol-induced NASH mice by either carbon tetrachloride administration or bile-duct ligation compared to untreated NASH mouse model [221]. LXR inverse agonists work by repressing de novo lipogenesis [222,223], therefore, ameliorating lipotoxic injury, which in turn could alleviate inflammation and fibrosis indirectly.

## 9. Conclusions

The evolutionary survival strategy adopted by early humans is responsible for its current phenotype. The abundance of food in modern times has discomposed metabolic flexibility resulting in developing metabolic syndrome. Nuclear receptors are key mediators in maintaining metabolic flexibility, and their dysregulation contributes to developing metabolic disorders, NAFLD, and cancers. Therefore, nuclear receptors represent promising therapeutic targets for these disorders. However, nuclear receptors regulate many different genes in various tissues, leading to engineered ligands presenting undesirable side effects, restricting their medical use. Because of this, research should focus more on the selective modulatory aspect of nuclear receptors in engineering their synthetic ligands.

## Figures and Tables

**Table 1:** Modulatory role of PPAR and LXR in metabolic processes.

Nuclear Receptors	Tissue Distribution	Endogenous Ligands	Synthetic Ligand	Metabolic Function
PPARα	Liver, heart, kidney, muscle	Saturated and unsaturated fatty acids, eicosanoids, 8(S)-HETE, and leukotriene B4	Fenofibrate Clofibrate Gemfibrozil Wy-14643	Fatty acid oxidation, lipoprotein metabolism, inflammatory control
PPARγ	Adipocytes, liver, kidney, macrophages	Saturated and unsaturated fatty acids, 15d-PGJ2, 15-HETE, 9-HODE, and 13-HODE	Rosiglitazone Pioglitazone Troglitazone Ciglitazone Farglitazar	Fatty acid storage, lipoprotein and glucose metabolism, inflammatory control
PPARβ/δ	Ubiquitously expressed, high in heart, muscle, liver	Saturated and unsaturated fatty acids, and 8(S)-HETE	GW-501516 GW0742 L-165041	Fatty acid oxidation, lipoprotein and glucose metabolism, inflammatory control
LXRα/β	Liver, adipose tissues, macrophages, ubiquitous (LXRβ)	Oxysterols	T-0901317 GW3695	Fatty acid and cholesterol metabolism, glucose metabolism, inflammatory control

## Data Availability

Not applicable.

## Abbreviation

ADD-1	Adipocyte differentiation and determinant factor
ACC	Acetyl-CoA carboxylase
AF-1/2	Activating factor 1, 2
ATGL	Adipose triglyceride lipase
BAT	Brown adipose tissue
CCL2	C–C motif chemokine ligand 2
C/EBP	CCAAT enhancer-binding protein
COX	Cyclooxygenase
DAG	Diacylglycerol
DBD	DNA-binding domain
DR1	Direct repeat 1
FASN	Fatty acid synthase
FFA	Free fatty acids
HSC	Hepatic stellate cells
HETE	Hydroxyeicosatetraenoic acid
HODE	Hydroxyoctadeca-9Z,11E-dienoic acids
HSL	Hormone-sensitive lipase
IκκB	Inhibitor of nuclear factor κB
IR	Insulin resistance
JNK	c-Jun N-terminal kinases
LBD	Ligand-binding domain
LPL	Lipoprotein lipase
LOX	Lipoxygenase
LTB4	Leukotriene B4
LXR	Liver X receptor
MGL	Monoglyceride Lipase
NAFLD	Non alcoholic fatty liver disease
NF-κB	Nuclear factor κB
PDG2	Prostaglandin G2
PNPLA3	Patatin-like phospholipase domain containing 3
PPAR	Peroxisome proliferator-activated receptor
PPRE	PPAR response element
PUFA	Polyunsaturated fatty acid
RXR	Retinoid X receptor
SFA	Saturated fatty acid
SCD-1	Stearoyl-CoA desaturase 1
SMRT SREBP-1	Silent mediator of retinoic acid receptor and thyroid receptor Sterol regulatory element-binding protein 1
TAG	Triacylglycerol
TLR4	Toll-like receptor 4
TGF-β	Transforming growth factor β
TNF-α	Tumor necrosis factor α
TZD	Thiazolidinediones
T2DM	Type 2 diabetes mellitus
WAT	White adipose tissue
15d-PGJ2	15-deoxy-Δ12,14-prostaglandin J2
